# Fungal Adenylyl Cyclase Acts As a Signal Sensor and Integrator and Plays a Central Role in Interaction with Bacteria

**DOI:** 10.1371/journal.ppat.1003612

**Published:** 2013-10-10

**Authors:** Yue Wang

**Affiliations:** 1 Institute of Molecular and Cell Biology, Agency for Science, Technology and Research (A*STAR), Singapore; 2 Department of Biochemistry, Yong Loo Lin School of Medicine, National University of Singapore, Singapore; The University of North Carolina at Chapel Hill, United States of America

Soon after birth, the human body establishes an intimate association with trillions of microbial cells belonging to numerous bacterial and fungal species [1]. These organisms coinhabit diverse microbial communities on cutaneous and mucosal surfaces such as the skin, the gastrointestinal tract, and the vaginal cavity [2]. Fungal and bacterial cells can interact in many ways, such as direct physical contact, secretion of toxins and signalling molecules, sharing or competing for metabolites and nutrients, and alteration of the environment [3]. These interactions can be antagonistic as well as mutually beneficial. Both bacteria and fungi have evolved sophisticated mechanisms to sense and respond to the presence and activity of other species nearby. In hospitals, fungi and bacteria are frequently isolated from the same site of infection. This raises the important question of whether fungi and bacteria interact in the process of infection, and whether the interaction dictates disease development and outcome, and if so, how they do so. This review summarizes recent discoveries in the study of signal sensing in the fungal pathogen *Candida albicans*. New findings support a model that adenylyl cyclases act as a hub of signal sensing and integration and may play a central role in bacterial sensing during fungal infection.

## cAMP Signalling Plays a Major Role in Regulating Cellular Responses to Environmental Signals and in Virulence in *C. albicans*

*C. albicans* is frequently found as a benign member of the normal microflora of humans. However, when conditions are favourable, it can cause a range of localized superficial infections such as rash and thrush in otherwise healthy people. But in immunocompromised patients, *C. albicans* can initiate life-threatening invasive infections with mortality rates as high as 75% [4]. Several traits of this fungus determine its virulence, including its ability to switch growth forms between yeast, pseudohyphae, and hyphae, expression of surface adhesion proteins, and secretion of proteolytic enzymes. Importantly, these traits are coregulated primarily by the cAMP signalling pathway [5]. A central component of this pathway is the cell's sole adenylyl cyclase Cyr1 that catalyses the synthesis of the second messenger 3′-5′-cyclic adenosine monophosphate (cAMP). In response to inducing signals, Cyr1 increases cAMP synthesis that in turn activates protein kinase A (PKA), leading to the expression of virulence genes. *cyr1Δ/Δ* mutants cannot undergo the yeast-to-hyphae transition and are avirulent [6]. Many external signals of distinct nature such as peptidoglycan (PGN), CO_2_, pH, and temperature are known to stimulate Cyr1 activity. Then, how does Cyr1 distinguish different stimuli or sense and integrate multiple ones to initiate a proper physiological response?

## Fungal Adenylyl Cyclases Are Large Proteins Containing Various Functional Domains Providing Multiple Points for Signal Sensing

**Figure 1:**
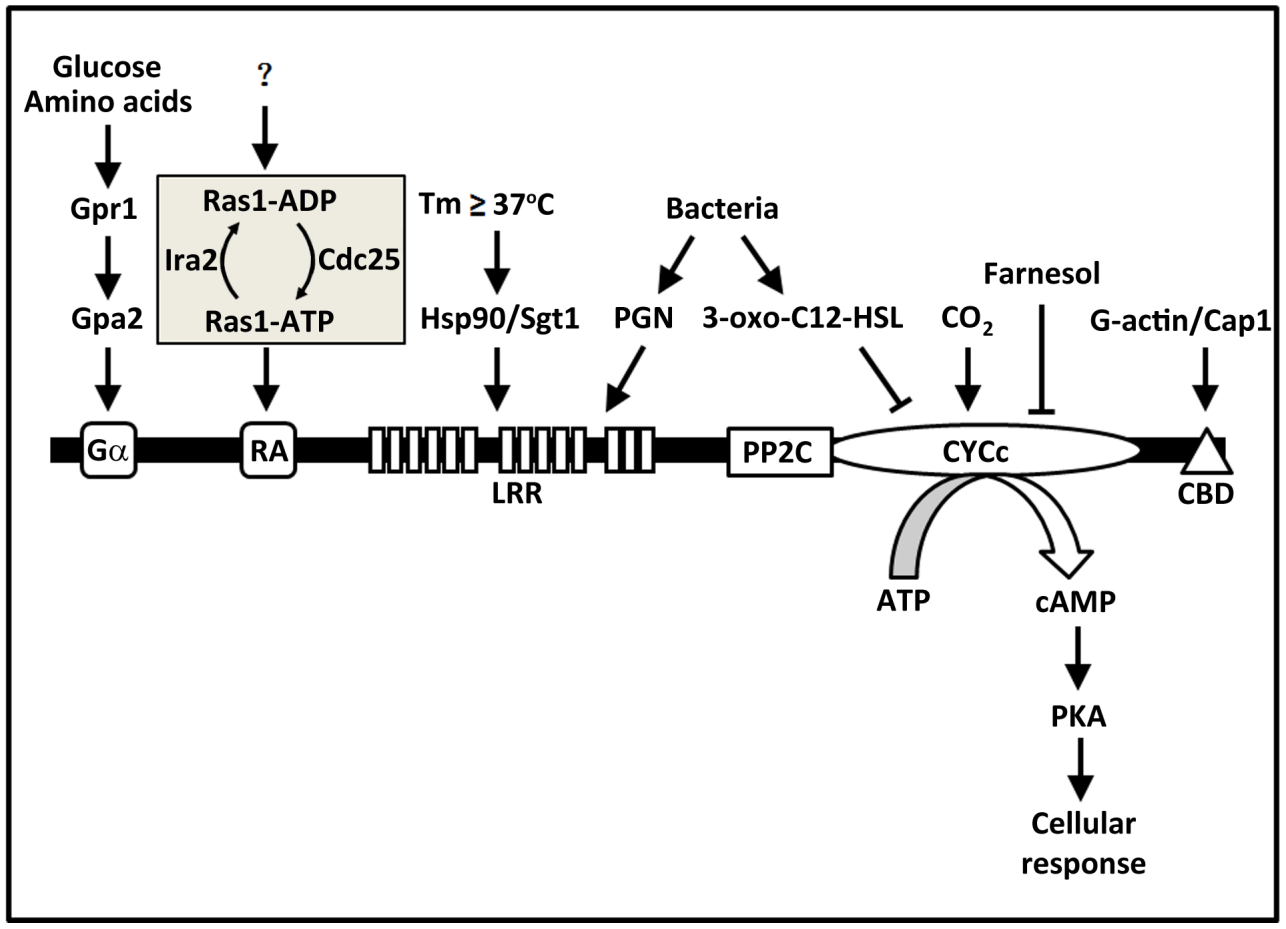
Fungal adenylyl cyclases contain multiple domains acting as sensors for a diverse range of signals. Evidence from many studies of the past decade or so supports a model in which fungal adenylyl cyclases function as a hub of signal sensing and integration. This figure illustrates all the conserved domains in fungal adenylyl cyclases and the external and internal signals each of the domains senses in *C. albicans* adenylyl cyclase Cyr1. For abbreviations and protein names, please refer to the text.

Fungal Cyr1s contain several highly conserved domains (Figure 1), from the N- to C-terminus, including a Gα domain, a Ras-association (RA) domain, a leucine-rich repeat (LRR) domain, a protein phosphatase 2C (PP2C) domain, a cyclase catalytic (CYCc) domain, and a Cap1 (cyclase-associated protein 1) binding domain (CBD). In *C. albicans*, the small GTPase Ras1, when in the GTP-bound form, activates Cyr1 by binding to the RA domain [7]. *ras1Δ/Δ* mutants are severely compromised in virulence and hyphal growth [8]. Yeast-2-hybrid experiments demonstrated direct association of Ras1 with the RA domain, and mutating conserved residues in RA was shown to block Ras1-Cyr1 interaction and prevent adenylyl cyclase activation [7]. Ras1 is thought to be activated by the guanine nucleotide exchange factor Cdc25 and inactivated by the GTPase-activating protein Ira2 [9]. Currently, it remains unclear as to what regulates the Ras regulatory module. The Gα domain of Cyr1 is thought to be the binding site for a G-protein α subunit Gpa2 that is activated by the G-protein-coupled receptor Gpr1 in response to amino acids and glucose [10], [11]. Deleting either *GPR1* or *GPA2* caused defects in hyphal formation on solid media in a cAMP-dependent manner. Although Gpa2 has been shown to bind to the Gα domain in fission yeast [12], such interaction has not been demonstrated in *C. albicans*. Cap1 is a well-known Cyr1-associated and G-actin-binding protein and is required for the activation of fungal adenylyl cyclase. *C. albicans cap1Δ/Δ* mutants are unable to increase cAMP synthesis upon hyphal induction, fail to undergo the yeast-to-hyphae transition, and are avirulent [13]. Recently, Zou *et al*. [14] isolated a tripartite protein complex containing Cap1, Cyr1, and G-actin, in which Cap1 serves as a bridge by binding to Cyr1 and G-actin through its N- and C-terminus, respectively. This complex can enhance cAMP synthesis in response to hyphal-induction signals *in vitro*, and in a manner dependent on Cap1 interaction with G-actin. This study suggests that Cyr1 may be able to sense the status of the actin cytoskeleton, a central player in polarized growth, and influence the cyclase activity. The Cap1 binding site has been mapped to the C-terminal tail of Cyr1 [15]. Raising temperature to above 37°C is normally required for the yeast-to-hyphae transition. The mechanism of temperature sensing was found to involve the heat shock protein complex Hsp90/Sgt1 through physical association with Cyr1 [16], [17]. In *S. cerevisiae*, Sgt1 was shown to influence cAMP signalling via direct interaction with the LRR domain of Cyr1 [18]. High CO_2_ concentration, found in many host niches, is another promoter of hyphal growth. Klengel *et al*. [19] discovered that CO_2_/bicarbonate activates Cyr1 by targeting the catalytic domain. In summary, four distinct domains of Cyr1 serve as sensor for signals as diverse as sugar, gas, temperature, and actin.

## *C. albicans* Cyr1 Directly Senses Bacterial PGN

Serum at 37°C is probably the strongest and physiologically relevant inducer of hyphal growth in *C. albicans*. Although the serum activity was first reported in 1956, the identity of the active molecule(s) remained as a mystery for decades. Recently, Xu *et al*. [20] discovered PGN fragments in serum fractions with high hyphal-inducing activity, and later confirmed that several muramyl dipeptides (MDPs), subunits of peptidoglycan, were indeed potent hyphal inducers. Mass spectrometry analysis detected ∼0.1 to 0.5 µM MDP in the blood of all healthy volunteers. As PGN is uniquely present in bacterial cell wall, the human microbiota is most likely the provider of PGN in the blood. Many bacteria are known to release a large amount of bioactive PGN subunits into the environment during cell wall remodelling [21]. Xu *et al*. [20] also demonstrated that MDP activates Cyr1 by binding to the LRR domain. Various mutations in the LRR domain completely abolished the hyphal growth induced by serum and MDP. This discovery has significant implications for the possible role of bacteria in *C. albicans* infection. As a commensal, *C. albicans* is constantly exposed to PGN fragments continuously released by trillions of bacterial cells. Although its effect on adenylyl cyclase activation may be balanced by other antagonistic factors, certain conditions may tip the balance in favour of *C. albicans* infection. For example, the use of broad-spectrum antibiotics, most of which inhibit PGN synthesis, may cause a massive release of PGN fragments. Together with antibiotic-associated colitis that damages the intestinal epithelium, PGN may enter the blood stream in large quantities, creating a window of opportunity for C. *albicans* to initiate systemic infection. This could be an important yet unappreciated factor underlying the high risk of candidemia in patients receiving high doses of broad-spectrum antibiotics.

## Farnesol and Bacterial Quorum-Sensing Molecules Inhibit *C. albicans* Hyphal Growth by Targeting the Catalytic Domain of Adenylyl Cyclase

Farnesol is a quorum-sensing molecule (QSM) produced by *C. albicans* that inhibits hyphal development and biofilm formation [22]. Early studies provided evidence suggesting that farnesol exerts its effect by interfering with the Ras/cAMP/PKA pathway [23], [24]. Hall *et al*. [25] later discovered that farnesol directly inhibits the cyclase activity of a truncated version of Cyr1 embracing the catalytic domain alone. Interestingly, the bacterial QSM 3-oxo-C12-homoserine lactone (HSL) secreted by *Paseudomonas aeruginosa* also inhibits *C. albicans* hyphal growth by a similar mechanism [25]. This mode of intertaxon chemical communication has important implications in the cause of microbial infections and ways to treat them. In the human microbiota, bacteria account for >99% of all microbial cells, which effectively checks fungal growth through secreting QSMs among other antagonistic mechanisms. However, disturbance of a microbial community by an antibacterial therapy may release the “brake” and create opportunities for commensal fungi such as *C. albicans* to initiate infection.

## Future Directions

Currently, the evidence is strong for fungal adenylyl cyclases as a coincidence detector [26]. To understand how their activity is kept low in the absence of stimuli and is turned on by different ligands either individually or in combination, structural elucidation of fungal adenylyl cyclases is urgently needed particularly in complex with interacting proteins and ligands. Also, the role of the LRR domain in signal sensing deserves more attention. A long LRR domain is present in most pattern recognition receptors of the innate immune system in animals and plants that recognizes a wide range of microbe-associated molecular patterns to elicit immune response [27]. So far, little can be found in the literature on the role of the LRR domain in fungal adenylyl cyclases. In *C. albicans* Cyr1, the LRR domain senses PGN. It is important to know whether the LRR domain in other fungal adenylyl cylases also plays a role in bacterial sensing. As members of the class III adenylyl cyclases, dimerization is required for catalysis [28]. However, it remains entirely unknown whether there is a dynamic and regulated monomer-dimer interconversion in fungal adenylyl cyclases. Equally elusive is their cellular localization. Answers to these questions may unveil additional dimensions for regulation.
